# Antibodies against *Leptospira* spp. in Captive Collared Peccaries, Peru

**DOI:** 10.3201/eid1305.060027

**Published:** 2007-05

**Authors:** Patricia Mendoza, Pedro Mayor, Hugo A. Gálvez, Manuel J. Céspedes, Ferran Jori

**Affiliations:** * Universidad Nacional Mayor de San Marcos, Lima, Peru; † Autonomous University of Barcelona, Bellaterra, Spain; ‡ Instituto Nacional de Salud, Lima, Peru; § Centre de Coopération Internationale en Recherche Agronomique pour le Développement, Montpellier, France

**To the Editor:** Leptospirosis is endemic to tropical South America and is a major public health problem for persons living in some regions of the Amazon Basin (*1**–**3*). For local inhabitants, the collared peccary (*Tayassu tajacu)* represents a major source of meat and income and is one of the most hunted species. As a result, several farms are attempting to produce captive collared peccaries (*4*). Although spirochetes have been isolated from bats, marsupials, and rodents in the Peruvian Amazon (*5*), local popular game animals have not been tested.

From May through December 2003, 96 collared peccaries from 4 experimental farms in 2 Amazonian provinces of Peru (Loreto and Ucayali) were surveyed for antibodies against *Leptospira* spp. Although the initial stock of each farm came from the wild, most animals had been born in captivity, remained on their respective farms, and had no contact with animals from the different farms. Blood samples were taken from animals that were born or maintained on the farm for ≥6 months, were in good physical condition, and showed no signs of disease. Samples that had been hemolyzed or otherwise contaminated were discarded, leaving optimal samples from 96 animals (sex ratio 1:1, 71% ≥1 year of age).

The microscopic agglutination test was performed with a panel of 24 antigens belonging to 17 serogroups of *Leptospira* spp. used for screening surveys at the National Leptospirosis Reference Laboratory. An additional distinct strain, obtained from a febrile human patient in the Peruvian Amazon and provisionally designated as Var10, was added (*2*). Serum samples were considered positive if they had 50% agglutination and titers >100 (*6*). Chi-square tests were used for statistical comparisons of sex and age; significance was set at p<0.05.

Among the screened samples, 64.6% reacted to 15 serovars (strains) that belong to 11 serogroups (Table). Seroprevalence did not differ significantly in relation to sex or age. Var10 was the most prevalent (56.2%) strain. This strain was isolated from a human patient in Iquitos (Loreto, Peru) and involved in recent outbreaks in the northern Peruvian Amazon (*2*); its taxonomic classification is pending. In terms of its distribution, 32 peccaries had positive results for Var10 only; 12 serum samples were reactive to >1 known serogroup. *Leptospira* sp. Var10 reacted mainly with serogroups Australis and Hebdomadis. Maximum titers were 6,400 for serogroup Tarassovi and 3,200 for Icterohaemorraghiae. High seroprevalence (15.6%) against serogroup Australis (serovar bratislava) has been reported in collared peccaries and in feral and domestic pigs (*7**,**8*)

**Table:** Prevalence of antileptospiral agglutinins per positive serogroup in captive collared peccaries, Peruvian Amazon, May 2003–Dec 2003*

Serogroup	Positive reactions no. (%)	Max titer	Loreto area		Ucayali area					
			BIOAM (n = 27)		Pucallpa Natural Park (n = 6)		San Juan (n = 52)		Club Divina Montaña (n = 11)	
			Positive reactions no. (%)	Max titer	Positive reactions no. (%)	Max titer	Positive reactions no. (%)	Max titer	Positive reactions no. (%)	Max titer
Total	62 (64.6)	6,400	27 (100)	6,400	1 (16.6)	100	27 (51.9)	3,200	7 (63.6)	100
Var10†	54 (56.2)	1,600	27 (100)	1,600	1 (16.6)	100	19 (36.5)	1,600	7 (63.6)	100
Australis	15 (15.6)	800	5 (18.5)	100	0	–	9 (17.3)	800	1 (9.1)	100
Hebdomadis	7 (7.3)	100	5 (18.5)	100	0	–	2 (3.8)	100	0	–
Icterohaemorrhagiae	4 (4.2)	3,200	2(7.4)	100	0	–	2 (3.8)	3,200	0	–
Autumnalis	4 (4.2)	100	3 (11.1)	100	0	–	1 (1.9)	100	0	–
Bataviae	4 (4.2)	400	2 (7.4)	200	0	–	2 (3.8)	400	0	–
Tarassovi	3 (3.1)	6,400	3 (11.1)	6,400	0	–	0	–	0	–
Djasiman	2 (2.1)	800	0	–	0	–	2 (3.8)	800	0	–
Grippothyphosa	2 (2.1)	800	0	–	0	–	2 (3.8)	800	0	–
Ballum	2 (2.1)	100	2 (7.4)	100	0	–	0	–	0	–
Canicola	1 (1.0)	100	0	–	0	–	1 (1.9)	100	0	–
Mini	1 (1.0)	100	0	–	0	–	1 (1.9)	100	0	–

*BIOAM, Biodiversidad Amazónica; Max, maximum; –, titer not applicable.  †Classification pending.

Seroprevalence on the farm in Loreto (n = 27) was 100%. At this farm, peccaries are kept near aquatic species and numerous ponds of stagnant water, which provide an ideal environment for the development of *Leptospira* spp. Because of recent human leptospirosis outbreaks in the area (*2*), 3 of the peccary caretakers were tested for antibodies against *Leptospira* spp.; their results were negative.

Although similar to animals described in previous reports (*7**,**9*), none of the sampled animals showed evidence of disease at the time of sampling; however, absence of clinical disease does not exclude the possibility of subclinical or past infections. Furthermore, the high prevalence of antibodies to multiple serotypes suggests a wide exposure to *Leptospira* spp. Despite reports that suggest the collared peccary could act as a reservoir for *Leptospira* spp. (*7**,**9*), the finding of high antibody titers in some individual animals could indicate that collared peccaries are incidental rather than reservoir hosts. However, the prevalences found at 4 distant farms also indicate that this species could play some role in the maintenance and spread of leptospirosis in the Amazon Basin.

Multiple titers to different serovars or serogroups in the same serum sample are common with serologic testing and difficult to interpret. Multiple titers can result from cross-reactions between different serovars or from true multiple infections (*10*). Regardless, serologic tests are only indicative of exposure to leptospires. Further efforts are necessary to isolate lepstospires from the urine or renal tissue of collared peccaries to confirm the presence of spirochetes and their potential dissemination into the environment.

Our findings indicate that persons who have contact with collared peccaries and their products, particularly animal caretakers, researchers, hunters, and game traders, are at risk for zoonotic disease (*3*). Because further wildlife production in the Peruvian Amazon is expected, movement of animals and high animal densities could increase the chances of spirochete transmission within and between the farms. Therefore, precautions should be taken to limit the potential risks for leptospirosis transmission to domestic animals and humans.
